# Effectiveness of Telepsychotherapy Versus Face-to-Face Psychological Intervention for Perinatal Anxiety and Depressive Symptomatology During COVID-19: The Case of an Italian Perinatal Psychological Care Service

**DOI:** 10.3390/brainsci15090963

**Published:** 2025-09-04

**Authors:** Beatrice Allegri, Giacomo Deste, Valeria Brenna, Emanuela Saveria Gritti, Linda Confalonieri, Alessandra Puzzini, Irene Corbani, Andrea Zucchetti, Umberto Mazza, Tamara Rabà, Mauro Percudani, Stefano Barlati, Antonio Vita

**Affiliations:** 1 Department of Mental Health and Addiction Services, Niguarda Hospital, Piazza Ospedale Maggiore 3, 20162 Milan, Italy; beatrice.allegri@ospedaleniguarda.it (B.A.); lindaconfalonieri@hotmail.com (L.C.); irene.corbani@gmail.com (I.C.); umbermazza@gmail.com (U.M.); tamara.raba@ospedaleniguarda.it (T.R.); mauro.percudani@ospedaleniguarda.it (M.P.); 2 Department of Clinical and Experimental Sciences, University of Brescia, 25123 Brescia, Italya.zucchetti001@studenti.unibs.it (A.Z.); antonio.vita@unibs.it (A.V.); 3 Department of Mental Health and Addiction Services, ASST Valcamonica, 25040 Esine, Italy; 4 Department of Humanities, University of Urbino Carlo Bo, 61029 Urbino, Italy; 5 Department of Mental Health and Addiction Services, ASST Spedali Civili of Brescia, 25123 Brescia, Italy

**Keywords:** pregnancy, COVID-19, depression, anxiety, telepsychotherapy, therapeutic alliance

## Abstract

Background: COVID-19 has limited pregnant and postpartum women’s access to mental health services, leading to the introduction of online interventions. Objectives: This study aims to compare the effectiveness of telepsychotherapy (i.e., psychotherapy provided through digital technology supporting real-time interactivity in the audio or audiovisual modality) with the one yielded by face-to-face interventions in treating perinatal depression and anxiety and to assess the therapist’s perceived alliance in both interventions. Methods: We collected anamnestic information and obstetrical risk factors for 61 women. We evaluated the effectiveness of face-to-face (N = 31) vs. telepsychotherapy (N = 30) interventions on depressive and anxiety symptoms at baseline (T0) and the end of treatment (T1) using the Edinburgh Postnatal Depression Scale (EPDS) and the State-Trait Anxiety Inventory (STAI-Y 1 and 2). We assessed the degree of alliance perceived by therapists with the Working Alliance Inventory (WAI-T). Results: Both groups showed significant decreases in depressive (EPDS face-to-face: T0 12.65 ± 5.81, T1 5.77 ± 4.63, *p* < 0.001; EPDS remote: T0 11.93 ± 5.24, T1 5.70 ± 4.46, *p* < 0.001; effect size: 0.002) and state anxiety (STAI-Y 1 face-to-face: T0 51.19 ± 13.73, T1 40.23 ± 12.86, *p* < 0.001; STAI-Y 1 remote: T0 51.10 ± 11.29, T1 38.00 ± 10.90, *p* < 0.001; effect size: 0.007//STAI-Y 2 face-to-face: T0 43.13 ± 12.11, T1 41.03 ± 13.06, *p* = 0.302; STAI-Y 2 remote: T0 44.20 ± 8.70, T1 39.30 ± 9.58, *p* = 0.003; effect size: <0.001) symptoms by the end of treatment. Women treated remotely also experienced a significant reduction in trait anxiety at T1 (*p* = 0.003). We found no significant differences in either symptomatology (EPDS; STAI-Y) between the two interventions at baseline or in the therapist-perceived alliance. Conclusions: Synchronous telepsychotherapy for perinatal depression and anxiety showed comparable treatment response to face-to-face interventions, with both modalities associated with significant symptom reduction and the establishment of a working alliance. These findings support the potential of telepsychotherapy as a valuable alternative when in-person services are not accessible, especially during emergency contexts.

## 1. Introduction

The outburst of the COVID-19 pandemic in December 2020 led to significant changes in people’s lifestyles, new challenges, and the emergence of interpersonal and social upheavals. Society’s daily routines changed from extreme hygiene to estrangement and restriction of freedom [1]. In this context, the perinatal period is critical, and pregnant women or mothers represent an at-risk population. The increased risk of infection (due to the natural suppression of the immune system) and the lack of knowledge about the possible vertical transmission of the virus to the fetus made this population more vulnerable and prone to depressive symptoms, stress, and anxiety, with consequences postpartum) [2]. In addition to biological vulnerability, several studies showed that COVID-19 worsened anxiety, stress, and depression during pregnancy and postpartum due to several social changes and lockdown restrictions [3,4,5,6]. It is recognized that a worsening of maternal mental well-being can lead to physical consequences during gestation, such as preeclampsia and hypertension, increased risk of miscarriage, and increased likelihood of preterm delivery [7,8]. In addition, giving birth during the pandemic was linked to increased dissatisfaction with delivery and a higher risk of postpartum depression [9]. Therefore, it is essential to recognize this vulnerable population’s needs and ensure incisive and consistent care.

Despite initial negative biases in the medical field [10,11], the advent of COVID-19 made therapists and clients more inclined to use new communication modalities in various therapeutic formats (e.g., synchronous telehealth psychotherapy) and with different populations [12,13] in order to adequately address emerging needs of remote interventions. Such modalities include telepsychotherapy, which is based on the use of digital technology supporting synchronous interactivity in the audio, audiovisual, or text modality. Telepsychotherapy is generally associated with user satisfaction and shows clinical outcomes similar to those of traditional face-to-face psychotherapy [14]. Additionally, it allows more people to be reached by promoting greater accessibility to treatment [15,16,17]. Therefore, psychotherapy provided remotely represents a popular and cost-effective choice, supported by empirical evidence of effectiveness. The literature demonstrates that online psychological counseling (including support via video, audio-treatment and online positive psychological programs) or psychotherapy services not only facilitate spontaneity but are effective in promoting psychological well-being by managing moments of transition and leading to an affective improvement in clients [18,19,20].

In terms of efficacy, several studies have supported the effectiveness of online interventions on various psychological issues. Based on a randomized controlled trial by Wagner and colleagues [21], differences in effectiveness between online and in-person therapy were not significant for the treatment of depression. Furthermore, the combination of two modalities (online self-help modules based on CBT principles and face-to-face interventions) was positively received by participants, who reported feeling more confident about treatment success due to integrating multiple tools [22]. Online therapy (internet CBT interventions) has also been shown to be effective for anxiety conditions such as panic disorders and agoraphobic symptoms [23], and studies on eating disorders also report symptom improvement with email therapy [24]. Italian national guidelines published by the Ministry of Health [25] identify the most significant benefits of telemedicine as equity of access to healthcare, improved quality of care, and increased continuity of care. Cognitive-behavioral therapy delivered via the Internet (CBT) is the most widely recognized psychological treatment for depression and anxiety in the general population [26].

However, in the perinatal context, which is still an evolving field, studies on the effectiveness of online interventions are quantitatively limited. One meta-analysis, including seven studies that evaluated six different psychological interventions delivered via the Internet, showed improvement in anxiety during the perinatal period from pre- to post-treatment (Hedges’ g = 0.76) and a smaller, non-significant, reduction in depressive symptoms (g ≤ 0.35) [27]. Similarly, a systematic review by [28] suggested that computer- or web-based interventions for depression, stress, and complicated grief during the prenatal, postpartum, or post-pregnancy periods may be effective. A review by [29] suggested that web-based therapies for perinatal depression administered in the postpartum period improved maternal mood.

In the perinatal setting, empirical studies have focused on iCBT. Based on a recent metanalysis iCBT significantly improves stress, anxiety, and depressive symptoms among postpartum women compared to Treatment as Usual (TAU) or control groups (Cohen’s d of 0.84, 0.36, and 0.63, respectively) [30]. A Randomized Controlled Trial [31] on a sample of pregnant women suffering from major depressive disorder found that the iCBT group had significantly lower levels of depressive symptomatology post treatment (g = 1.21) and were more likely to achieve a statistically reliable improvement (RR = 0.36; *p* = 0.004) [31]. However, more specific studies with a focus on interventions in the prenatal phase and that include control groups are needed. Furthermore, regarding the application of telemedicine to psychotherapy, few studies have specifically examined the construct of therapeutic alliance in the perinatal context, which is characterized by specific emotional vulnerabilities (anxiety, depression, identity changes) that require an empathic, co-constructed therapeutic relationship.

Therapeutic alliance can be defined as a collaborative relationship involving treatment tasks and goals, coupled with the emotional bond between therapist and patient. Therapeutic alliance is shown to be crucial for the success of psychotherapy according to Bordin’s classic definition [32]. According to Bordin’s pan-theoretical model [33], the working alliance consists of three key elements: agreement on tasks (the shared understanding of therapeutic activities that feel meaningful to the client), agreement on goals (the mutual definition of desired outcomes such as symptom reduction or personal growth), and the emotional bond (trust, respect, and attachment that provide a secure base for addressing difficult emotions). The working alliance is also a dynamic process that may strengthen or weaken throughout therapy, depending on the ongoing interactions between therapist and client.

Recent studies indicate that a robust working alliance can be established in videoconference-based psychotherapy, with levels comparable to face-to-face care [34,35,36]. In Internet-supported CBT for depression, the quality of the alliance is similar to that in in-person CBT, though effects on symptomatic outcomes may vary. Moreover, the alliance–outcome association in Internet-based interventions mirrors the one found in traditional psychotherapy (r ≈ 0.28) [37]. In the perinatal setting, initial evidence supports the feasibility of developing a therapeutic alliance through digital modalities. A pilot study in postpartum women found that telephone support within a web-based intervention enhanced engagement [38], while a scoping review confirmed the role of eHealth in fostering alliance through remote collaboration and active involvement [39]. Alliance appears crucial in this setting, where adherence is challenged by fatigue and emotional fluctuations, as it can improve engagement, reduce dropout, and promote long-term benefits for mother and child [39,40]. Given this background, the need for a more in-depth investigation of new ways to support mothers in the perinatal period arises.

The present study focuses on telepsychotherapy, specifically carried out using a desktop computer with a video camera or other instant communication devices (laptop, tablet, or smartphone). Due to the pandemic and its various stages of restriction, pregnant women and new mothers have been limited in several areas of life and have been unable to access mental health services as usual. Specifically, Northern Italy was greatly affected early on, and public and mental health services had to adapt to patients’ new needs and offer adequate and flexible care. Perinatal depression represents a specific clinical condition that differs from depression in the general population, as it often arises in women without a previous psychiatric history and is influenced by hormonal changes and unique psychosocial stressors. These distinctive features may affect treatment response, underlining the importance of evaluating the effectiveness of interventions specifically within this context.

Therefore, the present work aims to preliminarily explore the efficacy of face-to-face and telepsychotherapy modalities in treating perinatal disorders in an Italian public hospital, focusing mainly on depressive and anxiety symptoms during pregnancy and postpartum. Given the prevalence of interventions delivered remotely in recent years, we also aimed to assess the level of alliance perceived by therapists working in the perinatal setting based on the type of intervention delivered. In the light of the aforementioned literature, we expected no significant differences in the treatment response and level of alliance perceived by therapists across the face-to-face and telepsychotherapy modalities.

## 2. Material and Methods

The present study constitutes a prospective, non-pharmacological interventional investigation. The study has an observational nature and took place in a Treatment as Usual (TAU), naturalistic setting. No manualized interventions were provided. However, the researchers ensured that the therapeutic framework followed by the clinicians, the onset of the treatment and the number and length of sessions were similar across the telepsychotherapy and in-person treatment conditions. More details are provided in the Procedure Section.

### 2.1. Participants

Our study sample consists of 61 female participants referred spontaneously or who were referred by the Unit for Prevention and Treatment of Perinatal Disorders (Department of Obstetrics and Gynaecology of [blind for peer review]) from March 2020 to September 2022. The sample was divided into two groups: those who received face-to-face, in-person, clinical psychological treatment (N = 31) and those who participated in the clinical psychological treatment online through the Skype platform (N = 30). No patients presented with severe psychopathology in their history, and women were considered eligible for online psychological support in the absence of personality disorders, risk of psychosis, and PTSD. Participants were assigned to the two groups according to the mother’s logistic preferences and her skills with technology. In addition to the more stringent inclusion criteria for the online group described above, women could be admitted to the study if they were between 18 and 50 years old, had the reading skills and language comprehension skills needed to complete self-report questionnaires in Italian, and were pregnant or postpartum (up to the child’s first year). Furthermore, patients with severe systemic or neurological pathologies and the inability to give valid consent were excluded. All participants were provided with an information sheet about the purpose of the study, and they signed a written informed consent form to participate. Participants did not receive any reward.

### 2.2. Procedure

Since 2020, to fulfill the needs of mothers during pregnancy and postpartum, the Department of Mental Health at [blind for peer review] Hospital has expanded treatment options by introducing online psychological support for pregnant and postpartum mothers. These interventions are part of an innovative project to prevent and treat perinatal disorders in Italian and immigrant women. All participants were administered an ad hoc semi-structured interview to collect anamnestic information and gynecological obstetric risk factors. To address the research question regarding the respective outcomes and effectiveness of clinical psychological treatment interventions (i.e., face-to-face and online) on psychological aspects (i.e., level of depressive and anxious symptoms), patients in the pregnancy and the postpartum periods were compared and evaluated at two different time points (T0 and T1): at the beginning of the treatment (T0) and at its conclusion (T1). The average time that elapsed between the two time points for the two conditions (face-to-face and online) was, respectively, 7.2 ± 3.6 months and 9.27 ± 5.24 months. For mothers receiving online therapy, the psychotherapist conducted the assessment through the Skype platform, and the data collection procedure took place entirely online. In addition, an Italian version of a scale measuring perceived alliance at the end of treatment (T1) was administered to assess the degree of alliance between therapist and patient.

### 2.3. Interventions

At baseline, a psychologist from the clinical team met with the women who decided to participate in the study to collect information on anamnestic and risk factors. The clinician explained the importance of screening for depressive and anxious symptoms to prevent postpartum depression and its possible consequences on the well-being of both women and babies. All interventions aimed to increase confidence, hope, and comfort and to develop social, emotional, and functional support for the women involved in the study [41]. Interventions were delivered at medium intensity (one session per week when possible, or fortnightly). Initial sessions were devoted to identifying symptoms of depression and anxiety at baseline and establishing an alliance with the woman to create a “safe” interpersonal context. Next, the therapist and patient focused on problem areas and defined treatment goals. During subsequent sessions, the therapist addressed the interpersonal difficulties related to pregnancy and postpartum, including conflicts with partners or family, loss of social relationships, changes associated with pregnancy and childbirth, and other possible stressors, promoting improved coping and emotional regulation processes. In the final sessions, the therapist worked on enhancing the woman’s sense of competence as a mother and discussed plans for ending therapy. Pharmacological treatment was offered as an additional option for women who did not improve with psychological intervention alone and was monitored regularly until the psychopathological condition stabilized.

### 2.4. Therapists

The clinical team consisted of 4 female clinical psychologists, aged 30 to 40, all specializing in psychotherapy (cognitive, transactional, and Gestalt) and working at the Unit for Prevention and Treatment of Perinatal Disorders. Each therapist had over five years of experience in the perinatal hospital setting and was trained in the treatment of perinatal disorders. They also had comparable experience in providing remote psychological support, which became widespread after the COVID-19 pandemic. All therapists were actively involved in both treatment conditions, with a similar number of patients assigned to each of them.

### 2.5. Measures

We administered an ad hoc form to collect socio-demographic information and the presence of risk factors such as history of psychiatric disorders and previous psychological treatment, psychopathological family history, stressful life events that happened in the previous six months (financial problems, unemployment, change of job, change of residence, problems with partner, or family illnesses and deaths in the family), and pregnancy-related variables (e.g., physiological or pathological pregnancy, desired or unplanned pregnancy, parity, previous abortion). Subsequently, self-report scales (STAI-Y and EPDS) were administered to women at two different time points (T0–T1); these scales have been validated and are widely used in the perinatal context for assessing anxious-depressive symptoms and psychopathological risk factors. Such measures are routinely administered in the Service of Perinatal Psychology where the study took place to carry out screening and prevention activities.

#### 2.5.1. Anxiety Symptomatology Assessment

State-Trait Anxiety Inventory (STAI) [42,43] assesses anxiety symptoms and it consists of two questionnaires of 20 items on a Likert-type scale (1 to 4 points). The questionnaire evaluates state anxiety (i.e., anxiety perceived in the moment in which the questionnaire is filled in) and trait anxiety (i.e., anxiety that characterizes the individual’s personality regardless of the actual intensity of the situation). Scores for both the State Anxiety Scale and Trait Anxiety scales ranged from a minimum of 20 to a maximum of 80. A cut-off score of 40–50 indicates mild anxiety, 50–60 moderate anxiety, and over 60 severe anxiety. Internal consistency coefficients for the scale ranged from 0.86 to 0.95; test–retest reliability coefficients ranged from 0.65 to 0.75 over a 2-month interval [36]. Test–retest coefficients for this measure in the present study ranged from 0.69 to 0.89. The validated Italian version was used [44].

#### 2.5.2. Depressive Symptoms Assessment

The Edinburgh Postnatal Depression Scale (EPDS) [45,46] is a self-administered tool developed to assess the presence and severity of depressive symptoms in the pregnant and postpartum population. It consists of 10 items, and each question is scored from 0 to 3, with a maximum score of 30. A score of 13 or above is likely to indicate PPD (postpartum depression), and the individual should be evaluated further for diagnosis of depression. The EPDS focuses on two domains of negative affect: depressive symptoms and anxiety. The EPDS has a sensitivity rate of 90% and a specificity rate of 90% at an optimal cut-off score. The internal consistency, measured using Cronbach’s alpha coefficient, reached a value of 0.87, which indicates good reliability. The Italian version from Benvenuti et al., 1999, was used in the study [45].

#### 2.5.3. The Working Alliance Inventory—Therapist Version

The Working Alliance Inventory (WAI), developed by Horvath and Greenberg [47], is based on Bordin’s (1979) [33] theory and focuses on the collaborative relationship between therapist and patient. The therapist version (WAI-T) [48] measures the alliance from the clinician’s perspective with 36 items rated on a 7-point Likert scale (1 = never, 7 = always). The instrument includes three subscales: goal (i.e., agreement on therapy goals), task (i.e., agreement on tasks that are necessary to achieve treatment goals); and bond (i.e., emotional bond and trust between the therapist and the patient). The WAI-T is applicable across theoretical orientations and it assesses the early stages of therapy. Research confirms the WAI’s reliability and its strong correlation with therapy outcomes [49]. The Italian version by Lingiardi and Filippucci (2002) [50] was used. It is estimated that the Cronbach alpha of the WAI-T is approximately 0.93.

### 2.6. Statistical Analyses

Statistical analyses were performed using SPSS software Version 28. All demographic and clinical characteristics were entered into the statistical analysis for the whole sample. Descriptive analysis was conducted for each condition, including the patient’s sociodemographic and obstetric characteristics and psychological history. Descriptive statistics for continuous variables are given as means and standard deviation, whereas categorical variables (i.e., socio-demographic features, clinical and pregnancy-related variables) were presented as frequency (n) and percentages (%). The Chi-Square test was used to examine differences in the distribution of all categorical variables between two groups (face-to-face vs. online). Because not all participants completed every item, sample sizes may differ slightly for each analysis, as indicated in the following tables and text. Student’s *t*-test for independent data was used, when appropriate, to compare the mean values of continuous variables (EPDS, STAI-Y 1 and 2, WAI-T) among the two groups (face-to-face vs. online). The outcomes and effectiveness of psychological treatment interventions (face-to-face vs. online) were assessed directly through questionnaires (EPDS, STAI-Y 1 and 2) measuring anxiety–depressive symptomatology at T0 and T1. The degree of therapeutic alliance from the clinician’s perspective was assessed only at the end of the treatment (T1), and an independent *t*-test was performed between the two conditions. A paired *t*-test was performed to assess the mean difference between the two treatments in symptom improvement at T0 and T1. ANOVA was performed to assess if changes in the anxiety and depressive symptoms over time (T0 and T1) were different for those receiving face-to-face treatment or online psychological support.

## 3. Results

### 3.1. Socio-Demographic and Clinical Features of the Sample

A total of 61 pregnant women (mean age of 34.91 ± 5.32) were enrolled in the study. Of these, 31 women received a face-to-face intervention, while 30 received an online intervention for depressive and anxiety symptoms (synchronous telepsychotherapy). Most women were Italian (N = 54), and the average years of schooling were similar for both conditions (15.00 ± 4.43 vs. 15.33 ± 4.30). Most of the sample had stable job conditions (N = 54) and reported cohabiting with a partner (N = 56). A total of 68.85% of the women were primiparous, most presenting with an individual pregnancy (N = 59), and N = 4 underwent psychological treatment only postpartum. For women treated during pregnancy, the average of the start of treatment was 32.74 ± 9.27 (31.05 ± 10.2 vs. 34.47 ± 8.46) weeks. No significant difference was found in the univariate analysis concerning the different variables.

Complications considered during pregnancy, such as gestational diabetes, hyperemesis, and hypertension, affected 15 patients in the whole sample, and complications related to previous pregnancies involved 15% of the sample. Risk factors concerning problems with a partner, not being physically close to the family of origin, premenstrual syndrome, history of abortion, and the type of procreation (i.e., natural or medically assisted) did not differ between the two groups. Most women in both conditions planned their pregnancies (N = 49) and experienced spontaneous conception (N = 57). Most of the sample reported a satisfactory relationship with their partner (N = 45) and received family support (N = 44). N = 42 of women presented a history of psychopathology, but no statistically significant difference was found between the two conditions (N = 23 from the face-to-face group; N = 8 from the online group, *t* test: *p* = 0.360). N = 22 in the face-to-face group received psychological treatment in the past, and N = 15 of women in the online intervention. Only a minority of the sample, in both treatment conditions, were treated with pharmacological therapy and psychological support (N = 9 in the face-to-face group; N = 6 in the remote group). Only a minority of women presented with a history of abortion (N = 15). The demographic and clinical characteristics the participants in the two conditions are summarized in Table 1 and Table 2.

### 3.2. Sample Characteristics: Time Span Between Assessment at T0 and T1 and Symptomatology at the Baseline

The *t*-test for independent samples was performed to exclude differences in the time span (from T0 to T1) between the two conditions (Face-to-face: 7.2 ± 3.6 months; Online: 9.27 ± 5.24 months; *p* = 0.09). The results showed no significant differences, indicating that these two samples are comparable. The results also showed no significant differences in EPDS and STAI-Y 1 e 2 scores between the two types of interventions at the baseline (T0) (Table 3). All women were evaluated at both T0 and T1 for both conditions, and no dropout occurred.

### 3.3. Anxiety and Depressive Symptomatology Evolution

To determine whether the mean difference in anxiety and depressive scores between face-to-face and remote/online treatments at T0 and T1 was significantly different, a paired samples *t*-test was performed. The results confirmed a significant decrease in EPDS (*p* < 0.001) and STAI-Y 1 (*p* < 0.001) scores from baseline (T0) to the end of the treatment (T1) in both the face-to-face group and online group. Furthermore, women who received telepsychotherapy showed a significant decrease in trait anxiety symptoms (STAI-Y 2) scores at the end of the intervention (T1) (*p* = 0.003).

Subsequently, two-way repeated measures ANOVA was performed to determine the effect of treatment modality over time on anxiety and depressive symptoms. The normality of the distributions was checked by means of the Shapiro–Wilk test for each scale and time in the two groups. The homogeneity of the variances between the groups was examined using Levene’s test. The assumptions of the parametric tests were found to be satisfied for all variables excluding EPDS T1.

Regarding depressive symptoms (EPDS), the test did not show a significant interaction effect between time and the condition (*p* = 0.720). There was neither statistically significant interaction between the intervention and time on state anxiety symptoms (STAI-Y 1Y1, *p* = 0.668), and nor on trait anxiety symptoms (STAI-Y 2, *p* = 0.896). An investigation into effect size reveals, a negligible effect size in the context of the ANOVA. The EPDS exhibits an eta squared of 0.002, with Cohen’s f being 0.045. The STAI-Y1 demonstrates an eta squared of 0.007, with Cohen’s f being 0.084. The STAI-Y2 exhibits an eta squared < 0.001, with Cohen’s f < 0.001.

The results of the paired samples *t*-test and repeated measures ANOVA between T0 and T1 are shown in Table 3.

### 3.4. Therapeutic Alliance

Our results showed no significant difference between face-to-face and online/remote intervention in the therapist’s perceived alliance with the patient at the end of treatment. None of the subscales (client Bond, client Task, client Goal) showed a significant difference between the two conditions (Table 4). Both conditions showed an adequate degree of alliance.

## 4. Discussion

The health emergency caused by COVID-19 prompted research on the effectiveness of online interventions in the evolving field of perinatal care. Due to the pandemic, pregnant women and new mothers have been subjected to isolation, restricted freedom of movement, and reduced access to health services. Given the changes and adaptations of public mental health services to the new needs of a vulnerable population such as pregnant women, the main objective of the present work was to test the preliminary acceptability and effectiveness of online psychological treatment for perinatal disorders compared to standard face-to-face treatment, focusing mainly on depressive and anxiety symptoms in pregnancy and postpartum. Considering the literature and the significant uptake in multiple healthcare settings of online psychological support after the pandemic, we hypothesized treatment response to be equal to that of face-to-face support. The main result of the present study confirmed this hypothesis, showing that telepsychotherapy is as beneficial as face-to-face therapy to treat depressive and anxiety symptoms in women during pregnancy and postpartum. In our sample, we observed that anxiety and depressive symptoms tended to improve in both the face-to-face and online treatment conditions (7.2 ± 3.6 and 9.27 ± 5.24 months, respectively) compared with the baseline. These results are consistent with previous studies that compared face-to-face treatments with online interventions for various mental health problems [21,23,24,51,52] and with studies favoring the effectiveness of online psychotherapy in addressing mental health problems during COVID-19 [53,54,55]. Most importantly, our results are in line with studies conducted specifically in the perinatal period on anxiety and depressive symptomatology that report the effectiveness of Internet-based and remote interventions [29,56,57,58,59]. We attribute our findings and the effectiveness of online treatment to the several benefits that this type of intervention can provide during pregnancy and postpartum [60,61] and to the fact that patients enrolled in our study experienced personalized and interactive interventions, establishing relationships of mutual trust [62].

Several systematic reviews regarding remote interventions have shown that anxiety interventions are most effective when therapists provide support and guidance [63], an attitude that has been found to characterize online psychotherapy [64]. Such interventions during pregnancy and postpartum can provide easier access and reach mothers who are forced to stay at home with their newborn after birth (e.g., due to neonatal care, cesarean sections, and lack of practical support) or who present travel limitations due to physical or economic reasons. In addition, several studies have reported that patients are more likely to open up about their problems because the “virtual presence” is experienced as less threatening than the physical one. For some women, long-distance interventions provide a sense of anonymity, which makes them feel safer in revealing their symptoms [59]. More specifically, a new mother might refuse to undergo a psychological treatment for the fear of being labeled as a “bad mother” [65]. Therefore, long-distance interventions may be an option to offset what has been identified as the greatest perceived potential barriers to treatment, such as lack of time, stigma, and childcare issues [66].

Moreover, in our sample, there was a good proportion of mothers who presented almost clinical levels of state anxiety in both conditions; it may be that remote therapy addressed this type of anxiety, which often hinders the search for treatment (e.g., patients phobic or avoidant or with agoraphobia even exacerbated by the COVID-19 pandemic). However, our results did not show significant differences in improving trait anxiety symptoms in women who received the face-to-face treatment. We hypothesized that this result is explained by the fact that trait anxiety is a personality trait reflecting the frequency and severity of the emotional stress response and that this kind of anxiety is a more stable characteristic and has less to do with the situation the person is facing. The effect of the intervention on anxiety traits would probably be more evident with more prolonged treatment over time, which helps the person work on recognizing and modifying dysfunctional beliefs and cognitive biases [67]. In addition, during the perinatal period, a mother is more likely to miss a face-to-face session due to unforeseen circumstances that she cannot handle, and this aspect may be detrimental to improved anxiety functioning.

Another critical aspect investigated by the study was the degree of alliance, a key ingredient in therapy’s success as perceived by the therapist during psychological treatment. In our sample of women, the therapeutic alliance was assessed as being strong both in the online therapy condition and the face-to-face condition by the professional, and no differences emerged between the two. This result is slightly in contrast with data on telemedicine experiments, which have been characterized by various prejudices on the part of both patients and professionals [10,11,68] but is consistent with the most recent findings, which show that the therapeutic relationship in an online setting is consistently evaluated as being as positive and stable as that in a face-to-face setting, suggesting that a satisfactory working alliance can be well established in therapy delivered online [18,35,69]. It is conceivable that this change in perception is given mostly by the solid technological impulse that affected recent years; in particular, the COVID-19 pandemic contributed to the spread of online care services in various areas. Moreover, even if it does not consider the patient’s point of view, this information is essential as it addresses the paucity of information in the literature relating to the therapists’ experience. It should also be noted that in our sample all therapists were women belonging to the same age group, a factor that could have contributed to the homogeneity of the results and should be taken into account when interpreting these findings. From a clinical point of view, psychological support provided remotely is valuable as it enables the observation of a new mother in her home environment immediately after delivery and more frequently, which would not always be guaranteed in the face-to-face modality due to travel difficulties often experienced when taking care of a newborn. Telepsychotherapy also makes it easier for the physician to arrange expanded interventions with partners or family members who are often critical in the care of pregnant and postpartum women.

## 5. Conclusions

Our study offers preliminary evidence that teletherapy interventions provide a therapeutic benefit comparable to the one yielded by traditional face-to-face treatments in reducing depressive and anxiety symptoms during the perinatal period. While teletherapy emerged as a crucial resource during the COVID-19 pandemic, its benefits extend beyond emergency contexts, representing a valid and accessible alternative within perinatal mental healthcare. Nevertheless, further studies are needed to replicate these findings and to investigate possible differences in the therapeutic processes and mechanisms involved. As future generations grow increasingly accustomed to digital communication, the potential to form and sustain therapeutic relationships remotely must be taken into account. For this reason, professionals working in perinatal mental health should develop competence and confidence in using online tools to ensure timely, personalized, and effective support for women during and after pregnancy.

### Limitations and Future Directions

The present study has some limitations, including a small sample size due to data collection during the pandemic, when services were overloaded and mobility was limited. However, the low recruitment rate does not affect the representativeness of the sample, and the similarity in the demographic variables in the two groups suggests that changes in symptoms may be related to the type of intervention. The study did not include a randomized clinical sample or a control group not receiving treatment. Future research should focus on specific samples, include patients who drop out of treatment, and include follow-up evaluations and an assessment of the alliance perceived by the patient. This study represents pilot work. More extensive studies with structured interventions are needed to understand the long-term effects of psychological treatments at a distance and the implications for public health.

Secondly, although the EPDS, which we used to detect perinatal depression, is widely considered suitable for this purpose [46] it yields the shortcomings of a self-report method (e.g., potential recall bias or limitation to the individual’s awareness). Hence, future investigations should attempt to replicate the results of this study encompassing diagnostic methods such as, for instance, clinician-rated interviews.

## Figures and Tables

**Table 1:** Sociodemographic and pregnancy characteristics of women who received face-to-face and remote psychological support intervention.

Variables	Total (N, Mean ± SD) N = 61	Face-to-Face (N, Mean ± SD) N = 31	Remote (N, Mean ± SD) N = 30	*p* (*t*-Test, Chi-Squared)
Nationality (Italian/Not Italian)	54:7 (88.5:11.5)	26:5 (83.9:16.1)	28:2 (93.3:6.7)	0.246
Age	34.91 ± 5.32	34.11 ± 5.35	35.74 ± 5.25	0.235
School years	15.16 ± 4.33	15.00 ± 4.43	15.33 ± 4.30	0.767
Marital status (Cohabiting/Single)	56:5 (91.8:8.2)	28:3 (90.3:9.7)	28:2 (93.3:6.7)	0.668
Occupation (Unemployed/Employed/Student)	7:54 (11.5:88.5)	6:25 (19.4:80.6)	1:29 (3.3:96.7)	0.050
Primiparous (Yes/No)	42:19 (68.9:31.1)	19:12 (61.3:38.7)	23:7 (76.7:23.3)	0.195
Week of pregnancy	32.74 ± 9.27	31.05 ± 10.2	34.27 ± 8.46	0.266
Treated during pregnancy (Yes/No)	35:26 (57.4:42.6)	18:13 (58.1:41.9)	17:13 (56.7:43.3)	0.912
Treated during post-partum (Yes/No)	57:4 (93.4:6.6)	28:3 (90.3:9.7)	29:1 (96.7:3.3)	0.317
Twin pregnancy (Yes/No)	1:59 (1.7:98.3)	1:29 (3.3:96.7)	0:30 (0:100)	0.313
Assisted conception (Yes/No)	4:57 (6.6:93.4)	2:29 (6.5:93.5)	2:28 (6.7:93.3)	0.973
Planned conception (Yes/No)	49:12 (80.3:19.7)	20:11 (64.5:35.5)	29:1 (96.7:3.3)	0.002
Abortion (Yes/ No)	15:46 (24.6:75.4)	7:24 (22.6:77.4)	8:22 (26.7:73.3)	0.711
Complications (Yes/No)	15:46 (24.6:75.4)	8:23 (25.8:74.2)	7:23 (23.3:76.7)	0.823
Premenstrual syndrome (Yes/No)	22:39 (36.1:63.9)	12:19 (38.7:61.3)	10:20 (33.3:66.7)	0.662
Complications during other pregnancy (Yes/No)	9:12 (42.9:57.1)	5:9 (35.7:64.3)	4:3 (57.1:42.9)	0.350

**Table 2:** Clinical characteristics of women who received face-to-face and remote psychological support intervention.

Variables	Total (N, Proportion) N = 61	Face-to-Face (N, Proportion) N = 31	Remote (N, Proportion) N = 30	P (*t*-Test, Chi-Squared)
**Previous psychopathology** **(Yes/No)**	42:19 (68.9:31.1)	23:8 (74.2:25.8)	19:11 (63.3:36.7)	0.360
Anxiety	35:26 (57.4:42.6)	18:13 (58.1:41.9)	17:13 (56.7:43.3)	0.912
Mood Disorder	19:42 (31.1:68.9)	7:24 (22.6:77.4)	12:18 (40:60)	0.142
Eating Disorder	4:57 (6.6:93.4)	2:29 (6.5:93.5)	2:28 (6.7:93.3)	0.973
Personality Disorder	1:60 (1.6:98.4)	1:30 (3.2:96.8)	0:30 (0:100)	0.321
PTSD	2:59 (3.3:96.7)	2:29 (6.5:93.5)	0:30 (0:100)	0.157
OCD	1:60 (1.6:98.4)	1:30 (3.2:96.8)	0:30 (0:100)	0.321
Psychosis	0:61 (0:100)	0:31 (0:100)	0:30 (0:100)	0.252
**Previous Treatment** **(Yes/No)**	35:26 (57.4:42.6)	20:11 (64.5:35.5)	15:15 (50:50)	0.252
**Pharmacological treatment (Yes/No)**	15:46 (24.6:75.4)	9:22 (29:71)	6:24 (20:80)	0.413
**Stressful events** **(Yes/No)**	42:19 (68.9:31.1)	22:9 (71:29)	20:10 (66.7:33.3)	0.717
Illness/Bereavement	21:40 (34.4:65.6)	10:21 (32.3:67.7)	11:19 (36.7:63.3)	0.717
Job changes	13:48 (21.3:78.7)	9:22 (29:71)	4:26 (13.3:86.7)	0.134
Financial problems	6:55 (9.8:90.2)	3:28 (9.7:90.3)	3:27 (10:90)	0.966
Moving	7:54 (11.5:88.5)	2:29 (6.5:93.5)	5:25 (16.7:83.3)	0.211
Family conflicts	7:54 (11.5:88.5)	4:27 (12.9:87.1)	3:27 (10:90)	0.722
Marital problems	13:48 (21.3:78.7)	8:23 (25.8:74.2)	5:25 (16.7:83.3)	0.384
**Couple satisfaction** **(Yes/No)**	45:16 (73.8:26.2)	23:8 (74.2:25.8)	22:8 (73.3:26.7)	0.939
**Family Support** **(Yes/No)**	44:17 (72.1:27.9)	21:10 (67.7:32.3)	23:7 (76.7:23.3)	0.437

Note: PTSD, Post-traumatic Stress Disorder; OCD, Obsessive–Compulsive Disorder.

**Table 3:** Paired samples *t*-test and repeated measures ANOVA between T0–T1.

Variables	Intervention	T0 (Mean ± SD)	T1 (Mean ± SD)	*p* (*t*-Test)	*p* (Time × Group)	Effect Size (Eta Squared)	Effect Size (Cohen’s f)
**EPDS**	Face-to-face	12.65 ± 5.81	5.77 ± 4.63	**<0.001**	0.720	0.002	0.045
	Remote	11.93 ± 5.24	5.70 ± 4.46	**<0.001**			
**STAI-Y 1**	Face-to-face	51.19 ± 13.73	40.23 ± 12.86	**<0.001**	0.668	0.007	0.084
	Remote	51.10 ± 11.29	38.00 ± 10.90	**<0.001**			
**STAI-Y 2**	Face-to-face	43.13 ± 12.11	41.03 ± 13.06	0.302	0.896	<0.001	<0.001
	Remote	44.20 ± 8.70	39.30 ± 9.58	**0.003**			

Bold values indicate statistical significance.

**Table 4:** Differences between face-to-face and remote intervention in therapeutic alliance perceived by the therapist at the end of the treatment.

Variables	Total (Mean ± SD)	Face-to-Face (Mean ± SD)	Remote (Mean ± SD)	*p* (*t*-Test)
WAI-T_TOT	205.68 ± 21.66	204.94 ± 22.71	206.50 ± 20.83	0.784
WAI-client Bond	71.58 ± 6.54	71.77 ± 6.71	71.36 ± 6.47	0.809
WAI-client Goal	65.88 ± 9.43	65.03 ± 9.93	66.82 ± 8.93	0.469
WAI-client Task	68.20 ± 7.63	68.10 ± 7.97	68.32 ± 7.37	0.911

## Data Availability

The data supporting this study’s findings are available from the corresponding author upon reasonable request. The Authors chose not to display the study’s data on a public repository due to their high confidentiality and clinical nature, and to the relative vulnerability of the sample of participants involved (i.e., patients referred to a hospital Unit for Prevention and Treatment of Perinatal Disorders).
